# Germi-X herbal-based spray disinfects smartphone surfaces: implication on fomite-mediated infection spread

**DOI:** 10.1186/s13568-022-01369-y

**Published:** 2022-03-04

**Authors:** Acharya Balkrishna, Kanchan Singh, Swati Haldar, Anurag Varshney

**Affiliations:** 1 Drug Discovery and Development Division, Patanjali Research Institute, Roorkee-Haridwar Road, Haridwar, Uttarakhand 249405 India; 2 Department of Allied and Applied Sciences, University of Patanjali, NH-58, Haridwar, Uttarakhand 249405 India; 3 Special Centre for Systems Medicine, Jawaharlal Nehru University, New Delhi, 110067 India

**Keywords:** Herbal-based spray, Germicidal, Fomite-based infection, Hand and mobile sanitizer, Surface contamination

## Abstract

Inanimate objects/surfaces become fomites upon contacting infectious agents such as disease-causing bacteria, fungi and viruses. Smartphones are one of the most prominent among these fomites. COVID-19 pandemic has raised the awareness on mobile sanitization, as an active measure to curb fomite-mediated viral transmission. Available mobile sanitizers and ultraviolet (UV) ray mediated mobile sanitization have their own sets of pros and cons, often being less user-friendly. This study explored the germicidal efficacy of an herbal-based sanitizer, Germi-X spray, on hands and mobiles, through microbiological techniques of micro-broth dilution and Kirby-Bauer disc diffusion assay, thumb print assay and swab test. Notably, Germi-X spray was found to be 6–67% more effective against surface pathogens, like, *Staphylococcus epidermidis*, *Staphylococcus aureus*, *Pseudomonas fluorescens* and *Pseudomonas aeruginosa,* as compared to a very popular product in the Indian market, which was taken as a control for this study. The observed anti-bacterial activity of the spray from disc-diffusion assay suggests its greater surface retentivity as compared to the control. Germicidal potency of Germi-X spray, when used to sanitize hands, was found to be greater than 80%. There was ~ 17-fold reduction in microbial counts after sanitizing smartphones with Germi-X spray. The novelty of this study lies in providing experimental evidence for this herbal-based surface sanitizer in efficiently disinfecting one of the super contaminated fomite, the smartphones. In conclusion, having an herbal base with a high germicidal efficacy against surface pathogens, together with longer surface retention, Germi-X spray appears to be an eco-friendly and cost-effective sanitizer for the surfaces of electronic gadgets like smartphones.

## Introduction

Contact with contaminated surfaces is an important mode of transmission of infection not only in healthcare settings but within the community as well. Infected individuals deposit microorganisms on the surfaces of inanimate objects, like, smartphones, TV remotes, door knobs, hand-rails, counter tops, among several others, through frequent contact of these surfaces with their hands. Hands of an infected individual is actually a hot-bed of microbes and these hands touching different surfaces generate reservoirs of pathogenic microbes, which subsequently serve as vectors for transmitting infection. This indirect transmission of infection from an infected individual to others via inanimate surfaces is technically known as fomite-based transmission (Stephens et al. 2019; Olsen et al. 2020). Besides, bacterial and fungal infections, interestingly, fomites play a significant role in transmission of several viruses, like, norovirus (Jones et al. 2007; Bright et al. 2010), influenza A virus (Boone and Gerba 2005), picornavirus (Pappas et al. 2010), human rhinovirus (Gralton et al. 2015), rotavirus (Soule et al. 1999), different types human parainfluenza viruses (HPIV) (Boone and Gerba 2010; Stobnicka et al. 2018), human adenoviruses (Ganime et al. 2014) and even, severe acute respiratory syndrome (SARS) (Dowell et al. 2004) and middle east respiratory syndrome (MERS-CoV) (Khan et al. 2016) coronaviruses. In fact, fomite-based transmission of diseases has been found to be sufficient in the spread of many contagion, including that of SARS-CoV-2 (Kraay et al. 2018, 2021; Castaño et al. 2021). Noteworthy efforts are being made towards understanding the fomite-based transmission of diseases like Influenza through various approaches, such as, mathematical modeling and epidemiology (Zhao et al. 2012; Stephens et al. 2019). Fomites establish and sustain dormant microbial ecosystems, which come to life under conducive growth conditions, like the ones provided by host body (Gibbons et al. 2015; Chase et al. 2016; Hegarty et al. 2018; Hu et al. 2019). The efficiency of fomite-based transmission of an infection is directly proportional to the frequency of contaminated human touch the surface is exposed to (Chase et al. 2016). Hence, surfaces which are touched more often will serve as more potent fomites. So, an inanimate surface which is touched three to five thousand times a day, will actually be teeming with microbes. This is with reference to smartphones, the gadget, no matter, how much we try to avoid, has become an integral part of daily human life. With certain types of personal practices involving smartphones such as, carrying them to toilet, holding them in mouth when hands are not free or taking them to kitchen, have actually made this gadget not just a fomite but practically a reservoir of germs. In fact, a typical smartphone has 10 times more germs than that present on a toilet seat (Lo 2020). An interesting recent mathematical modeling study has confirmed the frequent sanitization of touch screen surfaces as an effective method of reducing transmission of infections (Di Battista et al. 2021). Of course, the ever-increasing awareness regarding safe use of smartphones, particularly, with the coronavirus disease 2019 (COVID-19) pandemic appearing in consecutive waves, that has increased the demand for smartphone sanitizers significantly. Smartphone sanitizers are mostly rubbing alcohols sold under different brand names. Currently, the use of smartphone sterilization using ultraviolet C (UVC) rays is rapidly becoming popular. However, both these options of sanitizing smartphones have their own demerits. Smartphones sanitization is a domestic cleansing process therefore, wearing gloves during sanitizing mobiles is not a very popular practice. The rubbing alcohol being popular as sanitizers if used very frequently can affect the skin. The persistent COVID-19 pandemic and rising anti-microbial resistance among microbes have actually encouraged very frequent sanitization of fomites, like, smartphones. This is likely to affect human health in several ways which are not yet foreseen. UV sterilization is suitable for exposed surfaces. The slots and crevices, with which smartphones are lavishly endowed remain safe from UV rays. Therefore, eco-friendly sanitizers with longer lasting effects are required. In this regard, herbal sanitizers could be promising. Thus, this study was designed to evaluate the germicidal efficacy of one such herbal sanitizer formulated and marketed by Patanjali Ayurved Ltd., under the trade name ‘Germi-X spray’. Besides *Aloe vera* gel and 70% v/v Ethyl alcohol, Germi-X spray contains aqueous extracts of *Ocimum sanctum* (English name: Holy Basil; Hindi name: Tulsi) and *Azadirachta indica* (English name: Indian Lilac; Hindi name: Neem), known for their potent antimicrobial effects (Rathod 2012). With herbal extracts and *Aloe vera* gel, Germi-X spary is likely to be eco-friendly with longer surface retention and enhanced effector time and thus, requiring less frequent applications. The following study was designed to first assess the germicidal activity of Germi-X spray in comparison to a commercially available popular domestic surface sanitizer through microbiological techniques of Kirby bauer (disc diffusion) and micro-broth dilution assays. Subsequently, its efficacy in removing microbes from hands (thumbs specifically) and smartphone surfaces was evaluated. The observations demonstrated that Germi-X spray not only inhibited growth of pathogenic bacteria in disc diffusion and micro-broth dilution assays, but also, effectively removed germ load from hands and smartphones.

## Methods

### Chemicals, bacteriological media, and bacterial strain

Chemicals used in this study were from Sigma-Aldrich (St Louis, MO, USA) unless otherwise mentioned. Bacteriological media were procured from Difco (BD Biosciences, San Jose, CA, USA). *Staphylococcus aureus* (MCC2408) and *Pseudomonas aeruginosa* (MCC3097) were procured from National Centre for Microbial resource (NCMR). *Pseudomonas. fluorescens* (MTCC2421) *and Staphylococcus epidermidis* (MTCC435) were procured from Microbial Type Culture Collection (MTCC), CSIR-Institute of Microbial Technology (Chandigarh, India). The test sample, GermiX Advanced Germ Kill Spray is a herbal germicidal spray, developed and manufactured by Patanjali Ayurved Ltd., Haridwar, India. Hereafter, it will be referred as Germi-X spray. This spray is composed of aqueous extracts of medicinal herbs like *Azadirachta indica* [common name: Neem (Hindi), Indian lilac (English)], *Ocimum sanctum* [common name: Tulsi (Hindi), Holy Basil (English)] and *Aloe barbadensis* [common name: Ghritkumari (Hindi), Aloe vera (English)] and ethyl alcohol. Germi-X spray (Batch # AAKU002, Manufacturing: November 2020, Expiry: 24 months from the date of manufacturing) was procured from Patanjali Megastore, Haridwar and was manufactured as per the license no. Uttra.Ayu-181/2009 issued by Licensing Authority, Ayurvedic and Unani Services, Uttarakhand, Dehradun, India. The comparative control used in this study for the microbiological tests is a popular marketed domestic sanitization spray (Brand name: Lifebouy, manufactured by Hindustan Unilever Limited, Mumbai, India) available in India.

### Antibacterial assay

#### Screening of anti-bacterial effect through Kirby Bauer or disc diffusion assay

100 µl overnight cultures of *S. epidermidis*, *S. aureus*, *P. fluorescens* and *P. aeruginosa* each containing 10^8^ cells were uniformly spread over Muller Hilton Agar (MHA) plates. Sterile filter paper discs of 5 mm diameter pre-soaked with 50 µl Germi-X spray or control spray were then placed on these plates. Two discs, one with Germi-X spray and other with the control spray were placed on each plate before incubation at 37 °C for 24 h. Post-incubation diameters of the clear bacteria free zones were measured.

#### Determination of inhibitory concentration through microbroth dilution method

The antibacterial potency of Germi-X spray was evaluated through microbroth dilution method as per the standard guidelines of the Clinical and Laboratory Standards Institute (CLSI, 2015) against *S. epidermidis*, *S. aureus*, *P. fluorescens* and *P. aeruginosa*. Subsequent to series of two-fold serial dilutions of the test article over a range of 0.08 to 100 volume/ volume percent (% v/v), 100 μl of desired bacterial suspension in Muller Hilton Broth (MHB) [containing 10^8^ colony forming units (CFU)/ml] was added to an equal volume of each dilution of Germi-X spray in 96-well plates. After a 24 h incubation at 37 °C with 180 rpm shaking, absorbances were measured at 600 nm in a microplate reader (Envision, Perkin Elmer). An identical concentration range of control spray was used for comparison. Percent bacterial growth inhibition by Germix spray was calculated using the following equation:

$$\% {\text{ Bacterial Growth Inhibition }} = \, \left[ {\left( {{\text{A}}_{{\text{c}}} - {\text{A}}_{{\text{t}}} } \right)/{\text{A}}_{{\text{c}}} } \right]*{ 1}00$$

where A_c_: Absorbance of the control; A_t_: Absorbance of test sample (Germi-X spray/standard).

Data is represented graphically as mean ± SE of percent (%) inhibition calculated from three independent experiments using Graphpad Prism (version 7.0) (SanDiego, CA, USA). Minimum inhibitory concentrations sufficient for 30 (IC_30_), 50 (IC_50_), 70 (IC_70_) and 90 (IC_90_) % inhibition of Germi-X spray and standard were determined using inbuilt data analysis options of Graphpad Prism software. Dose response curves for Germi-X spray and standard for all bacterial strains were also plotted indicating these inhibitory concentrations.

#### Determination of germicidal efficacy on skin through thumb print assay

Thumb impressions from 75 volunteers were taken on sterile tryptic soya agar plates with and without sanitizing with Germi-X spray. The volunteers in the group without Germi-X spray sanitization, were requested to wash their hands with water before giving thumb prints. Thumb prints were collected before and after washing/sanitization. The plates were then incubated under aerobic conditions in incubator at 37 °C for 24 h. Germicidal potency was calculated by counting the number of visible microbial colonies present before and after application of spray or water using the following formula:

$${\text{Germicidal Potency }}\left( \% \right) = \, \left[( {{\text{BBW}} - {\text{BAW}}} \right)/{\text{BBW}}]*{1}00$$

where, BBW stands for bacterial load before wash/sanitization, and BAW stands for bacterial load after wash/ sanitization with Germi-X spray.

#### Determination of germicidal efficacy against smartphone surface microflora

The study was conducted on 50 mobile phones voluntarily offered by colleagues at Patanjali Research Institute, Haridwar, India. However, participants were not informed beforehand about the date of sampling to ensure random unbiased sampling. Using sterile cotton swab soaked in normal saline, microflora was collected from the entire exposed surface of each smartphone and immediately spread on Tryptic Soya Agar (TSA) plates and incubated at 37 °C for 24 h. The number of viable microbes present before and after sanitization with Germi-X spray were counted.

## Results

### Germi-X spray effectively eliminated pathogenic bacteria and created clear zones-of-inhibition

**Fig. 1:**
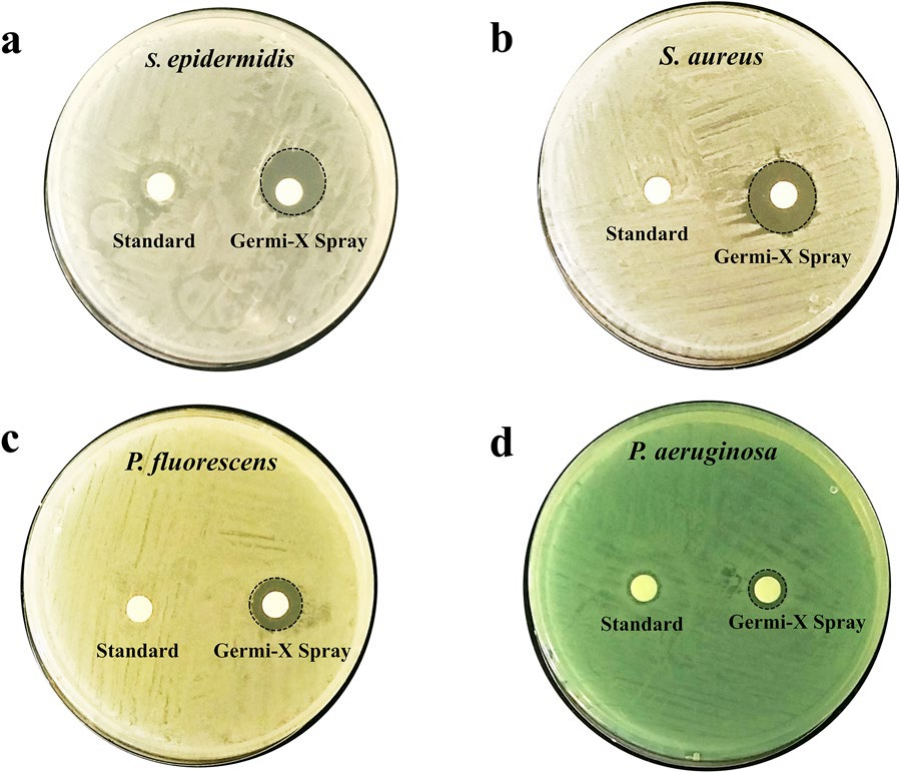
Kirby Bauer disc diffusion assay showing anti-bacterial activity and prolonged surface retention of Germi-X spray. **a**–**d** Prolonged surface retention of Germi-X spray is depicted with representative digital images of *S. epidermidis* (**a**), *S. aureus* (**b**), *P. fluorescens* (**c**) and *P. aeruginosa* (**d**) showing the zones of inhibition (demarcated with black broken circles) around the discs soaked with Germi-X spray. Discs soaked in standard germicidal spray did not form any such bacterial growth free surrounding zones

The preliminary assessment of the germicidal effect of Germi-X spray was conducted through the classical Kirby Bauer or disc diffusion assay. Sterile filter paper discs were soaked in either Germi-X or standard spray and placed on bacterial lawns of *S. epidermidis*, *S. aureus*, *P. fluorescens* and *P. aeruginosa*. For proper comparison and to reduce experimental variability due to different bacterial lawns, discs with Germi-X and standard spray were both placed on the same agar plate for each pathogen (Fig. 1a–d). The standard did not form any zone-of-inhibition on any of the four bacterial lawns, whereas, discs soaked with Germi-X spray developed distinct bacteria-free clear circular zones around themselves over all the bacterial lawns. These clear zones were formed due to removal of bacteria from those areas. Their appearances around the Germi-X-soaked discs implies that Germi-X spray was capable of eliminating bacteria around itself. The diameters of the zones-of inhibition give a relative idea of the extent of ensuing bacterial elimination from the zone. The biggest zone-of-inhibition (16 ± 1.5 mm) (Fig. 1a) was observed in case of *S. epidermidis*, followed by those formed over *S. aureus* (Fig. 1b), *P. fluorescens* (Fig. 1c) and *P. aeruginosa* (Fig. 1d) lawns, in that order (Table 1). Taken together, these observations, clearly demonstrate that Germi-X spray was effective against the pathogenic, *S. epidermidis*, *S. aureus*, *P. fluorescens* and *P. aeruginosa*. It was indeed intriguing to notice that despite being an alcohol containing sanitizer, the standard spray failed to develop any zone-of inhibition. Perhaps, the standard sanitizer, being rich in alcohol evaporated instantaneously and was therefore, was not retained on the disc for sufficiently long to exert its bactericidal effect. On the other hand, presence of *Aloe vera*, rate of evaporation of Germi-X spray was reduced and its surface retention prolonged. This increased its bioavailability and eventually improved its germicidal effect.

**Table 1:** Diameters of zones-of-inhibition formed by germicidal sprays on lawns of different bacterial pathogens

Bacterial pathogen	Diameter of zone-of inhibition (mean ± SEM) (mm)	
	Germi-X spray	Standard
*Staphylococcus epidermidis*	15.97 ± 0.09	–
*Staphylococcus aureus*	13.03 ± 0.20	–
*Pseudomonas fluorescens*	11.13 ± 0.09	–
*Pseudomonas aeruginosa*	8.23 ± 0.15	–

### Germicidal efficacy of Germi-X spray

**Fig. 2:**
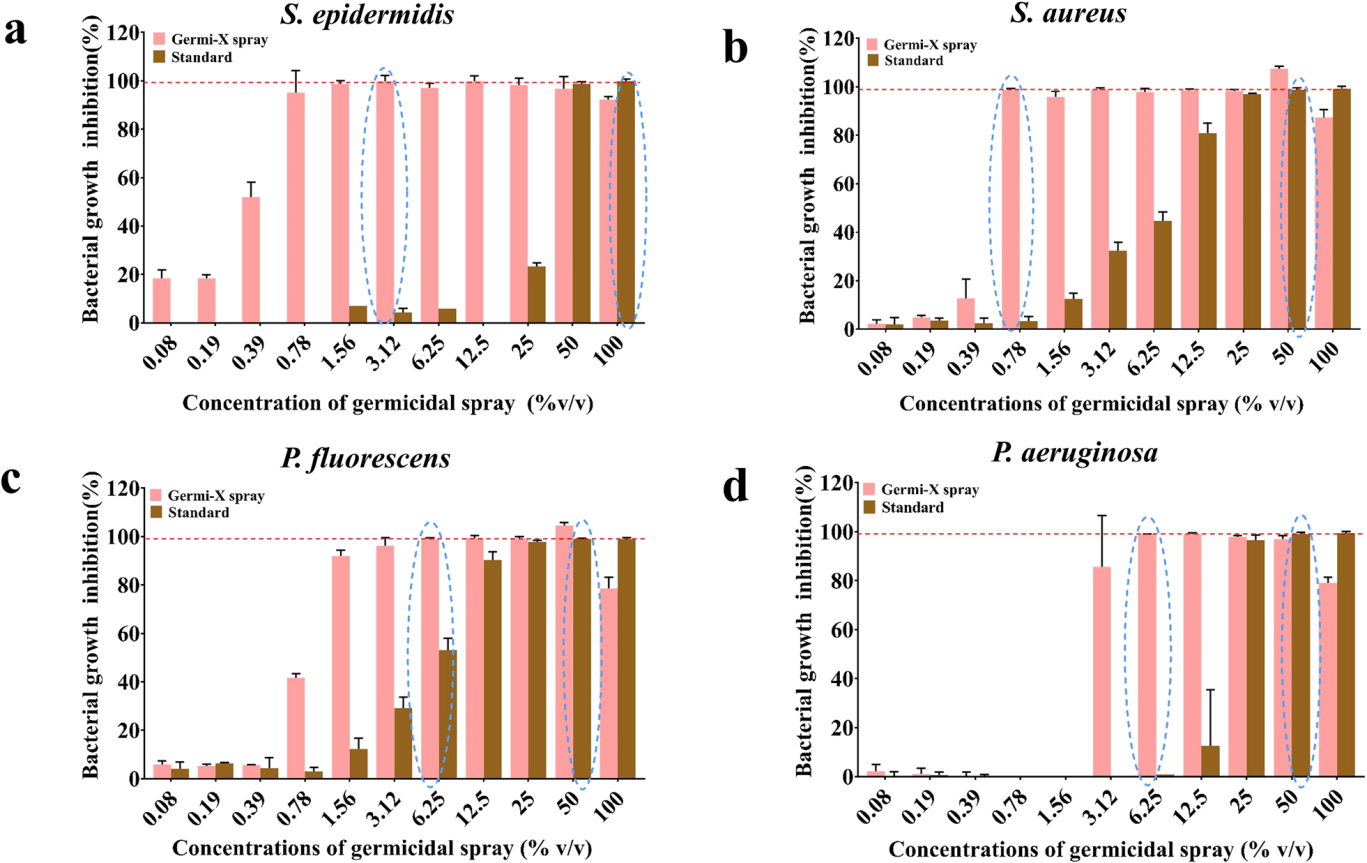
Germicidal effect of Germi-X spray on different surface pathogenic bacteria. **a**–**d** Grouped column graphs representing comparisons between % bacterial growth inhibition exhibited by Germi-X spray and standard germicidal spray against *S. epidermidis* (**a**), *S. aureus* (**b**), *P. fluorescens* (**c**) and *P. aeruginosa* (**d**). The red broken lines are used to indicate 99% of the respective bacterial growth and blue open oblongs are used to mark the concentrations of Germi-X spray and standard at which 99% inhibition of respective bacterial growth was achieved

**Fig. 3:**
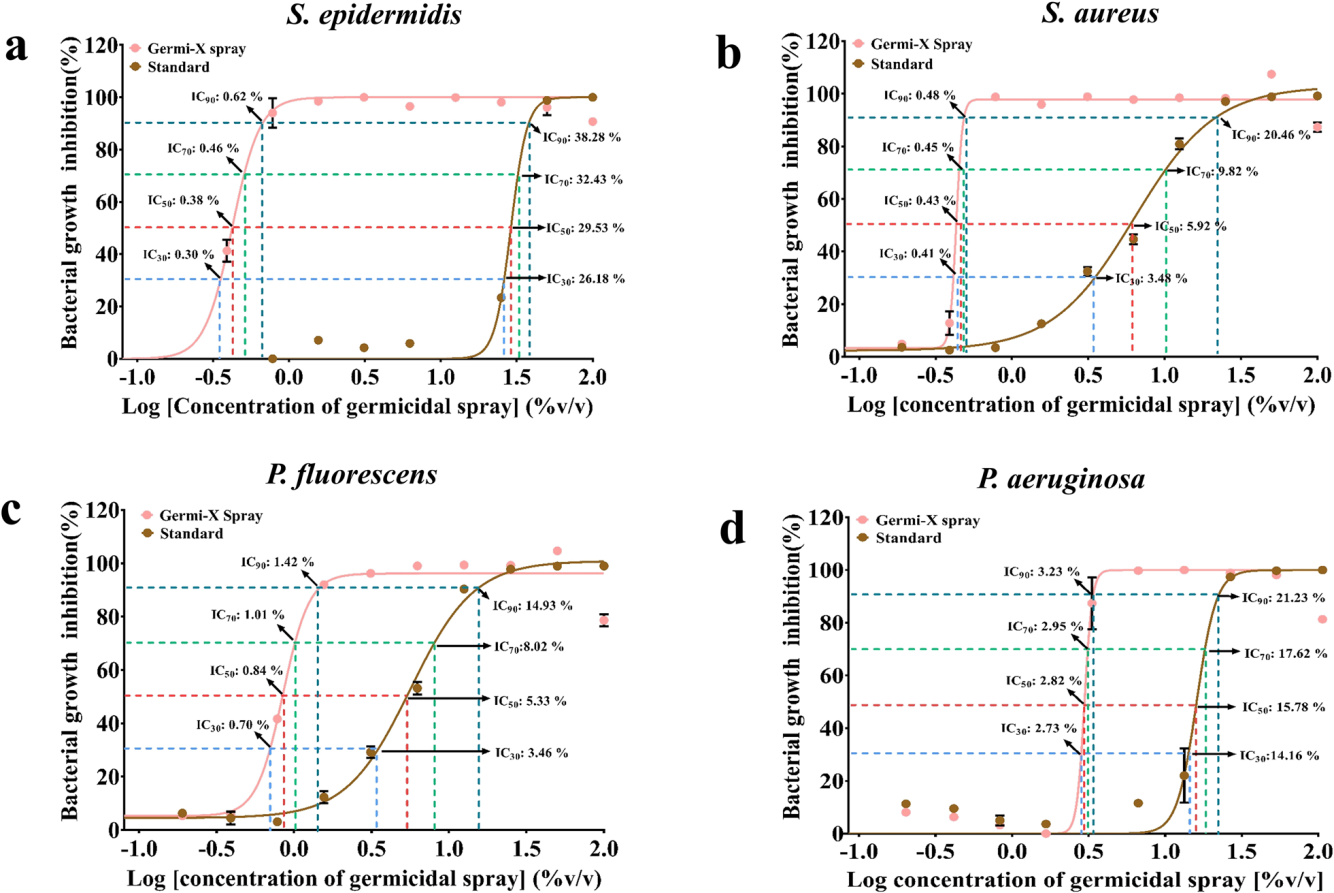
Germicidal potency of Germi-X spray. **a**–**d** Dose–response curves showing dose-dependent inhibitory effect of Germi-X spray and standard on the growth of *S. epidermidis* (**a**), *S. aureus* (**b**), *P. fluorescens* (**c**) and *P. aeruginosa* (**d**) Concentrations responsible for 30 (IC_30_), 50 (IC_50_), 70 (IC_70_) and 90 (IC_90_) % growth inhibitions, as determined through non-linear regression analysis, are mentioned

Germicidal efficacy of Germi-X spray was evaluated through the micro-broth dilution method. Germi-X spray inhibited the growth of all the four pathogenic bacteria, namely, *S. epidermidis*, *S. aureus*, *P. fluorescens* and *P. aeruginosa* more efficiently than the standard spray. 90 and 99% growth of *S. epidermidis* was evident respectively, at 0.78 and 1.56% volume/volume (v/v) dilution of Germi-X spray (Fig. 2a). Likewise, 99% growth of *S. aureus* was inhibited at 0.78% v/v dilution of Germi-X spray (Fig. 2b). Comparable inhibitions with the standard spray for *S. epidermidis* and *S. aureus* were achieved at 50% v/v dilution which was respectively, 32 and 64 times concentrated than the effective concentrations of Germi-X spray against these bacteria (Fig. 1a, b). Germi-X spray was slightly less aggressive against the other two pathogens, *P. fluorescens* and *P. aeruginosa*, when compared to *S. epidermidis* and *S. aureus*. Nevertheless, growth inhibitory effects of Germi-X spray of *P. fluorescens* and *P. aeruginosa* were significantly more pronounced relative to the standard spray. Germi-X spray inhibited 99% growth of *P. fluorescens* (Fig. 2c) and *P. aeruginosa* (Fig. 2d) at 6.25% v/v dilution which was respectively, 4 and 8 times more concentrated than those effective for comparable growth inhibition of *S. epidermidis* (1.56% v/v) and *S. aureus* (0.78% v/v). For similar growth inhibition of *P. fluorescens* and *P. aeruginosa*, 50% v/v dilution of the standard was sufficient, which was eightfold more concentrated than corresponding effective concentration of Germi-X spray (Fig. 2c, d). Improved germicidal potency of Germi-X spray was clearly evident from the dose response curves (DRCs) against each of the four pathogenic bacteria included in this study (Fig. 3a–d) and the enlisted IC_30-90_ values for Germi-X spray and standard in Table 2. Clearly, these observations showed that the standard spray was significantly less effective on *S. epidermidis*, *S. aureus*, *P. fluorescens* and *P. aeruginosa*. Therefore, it was deliberately excluded from subsequent studies.

**Table 2:** IC_30-90_ values of Germi-X spray and standard against different bacterial pathogens

Inhibitory concentrations (% v/v)	*Staphylococcus epidermidis*		*Staphylococcus aureus*		*Pseudomonas fluorescens*		*Pseudomonas aeruginosa*	
	Germi-X spray	Std	Germi-X spray	Std	Germi-X spray	Std	Germi-X spray	Std
IC_30_	0.30	26.18	0.41	3.48	0.70	3.46	2.73	14.16
IC_50_	0.38	29.53	0.43	5.92	0.84	5.33	2.82	15.78
IC_70_	0.46	32.43	0.45	9.82	1.01	8.02	2.95	17.62
IC_90_	0.62	38.28	0.48	20.46	1.42	14.93	3.23	21.23

### Effectivity of Germi-X spray as a hand and smartphone sanitizer

**Fig. 4:**
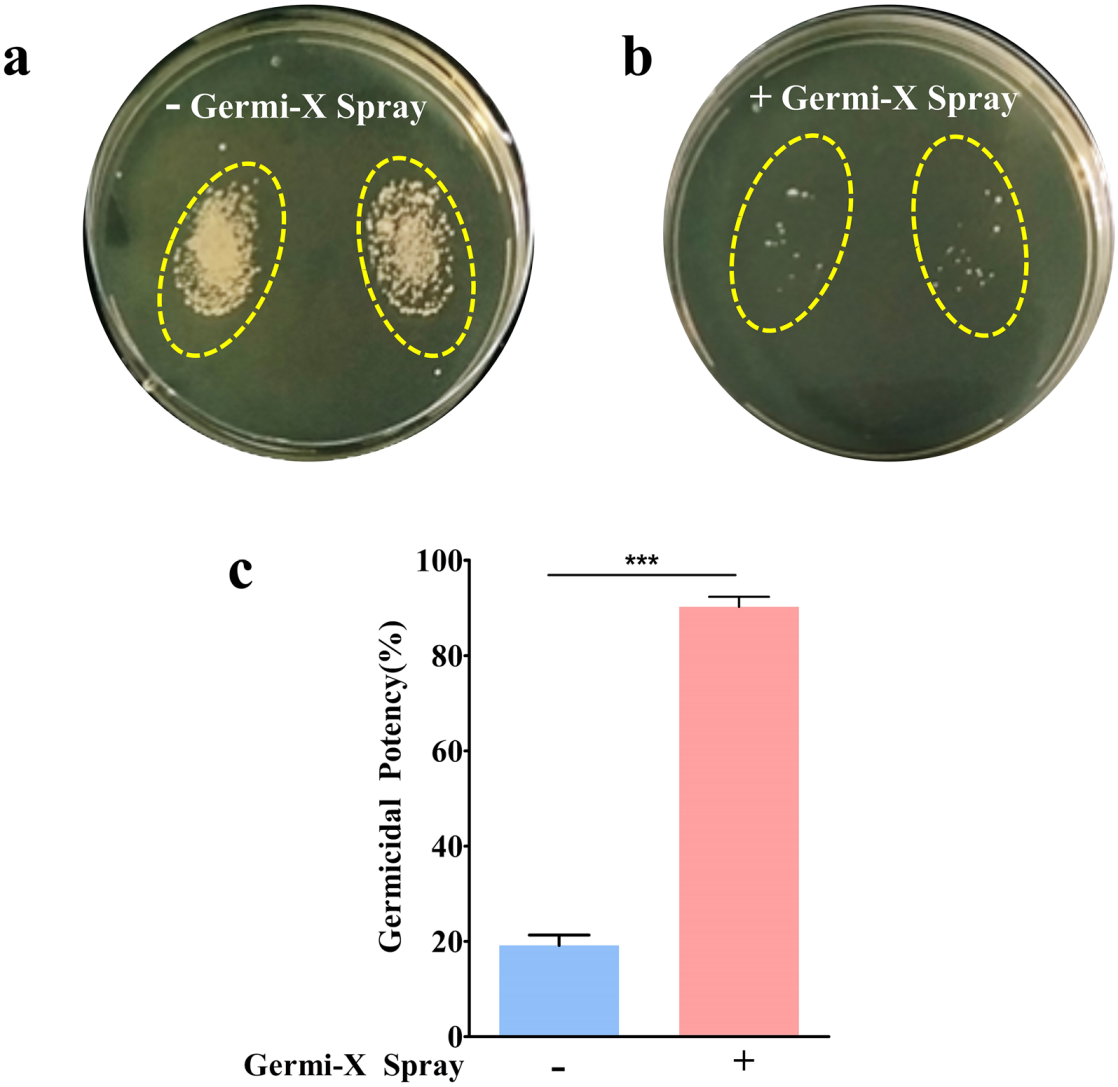
Sanitizing efficacy of Germi-X spray. **a**, **b** Representative digital images of bacteriological plates with thumb prints from the same volunteer without (**a**) and with sanitization (**b**) with Germi-X spray. **c** Sanitization efficacy of Germi-X spray is represented as % germicidal potency bar graph. Statistical significance was determined through *Student’s t-test* and observation represented as ***p < 0.001 for the difference between sanitization without and with Germi-X spray

In real life situation, fomite-induced pathogen transmission results from frequent contacts of inanimate surfaces with contaminated hands. Therefore, effectivity of Germi-X spray in removing germs from hands was evaluated through an ingenious method (Balkrishna et al. 2020). In this method, thumb prints of volunteers without and with sanitization with Germi-X spray were collected on nutrient rich agar plates, which were then incubated under conditions conducive for microbial growth (Fig. 4a, b). Subsequently, colonies were enumerated and germicidal potency determined. Since, no suitable standard was available, therefore, prints from thumbs rinsed with water were considered as a control Germi-X spray showed 90% potency in killing germs on the thumbs whereas, washing thumbs with water could remove only ~ 18% of the germs (Fig. 4c). Thus, Germi-X spray could remove germs from thumbs 5 times more effectively than by simple washing with water. To sum it up, Germi-X spray is effective in removing germs from hands, suggesting a higher likelihood of reduced fomite-induced pathogen transmission. Regardless of this observation, evaluation of direct effect of Germi-X spray on the germ loads of fomites is warranted. So, the efficacy of Germi-X spray in removing germs from smartphones (one of the most common fomites) was evaluated subsequently.

**Fig. 5:**
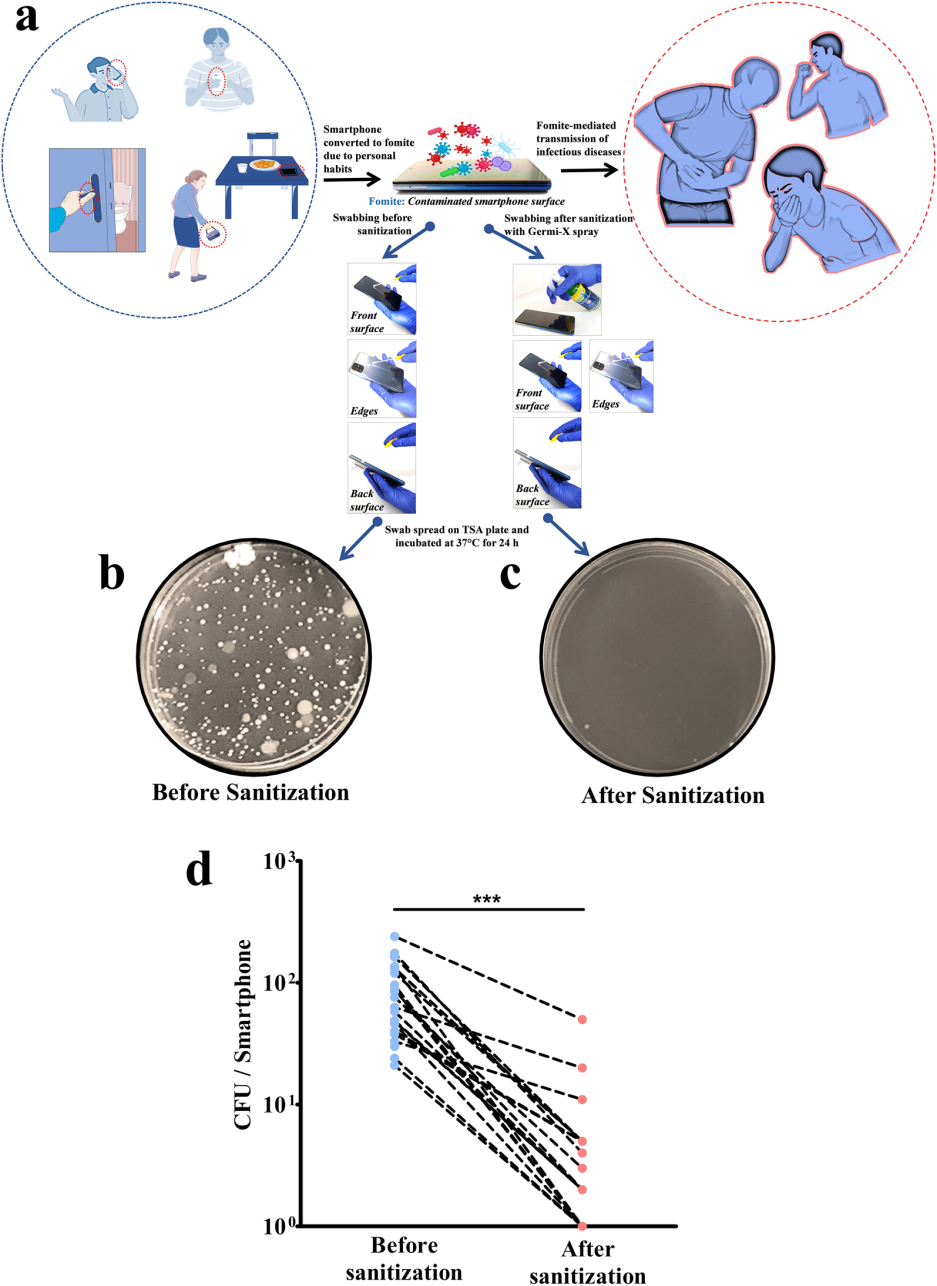
Effectivity of Germi-X spray as a smartphone sanitizer. **a** Pictorial depiction of acquisition of germs on inanimate surfaces thereby forming fomites and their subsequent role in transmitting diseases. The illustration also summarizes the experimental steps followed for collecting swab samples from smartphones before and after sanitization with Germi-X spray. **b**, **c** Representative digital images of bacteriological plates showing the growth of microflora in the swab sample collected from smartphones before sanitization with Germi-X spray (**b**) and its lack thereof after sanitization (**c**). **d** Paired line graphs depicting the reduction in germ load after sanitization of smartphones with Germi-X spray. The statistical significance between the average CFUs in the two groups is determined through *Students’s t-test* and indicated as ***p < 0.001

Swab samples from smartphones of volunteers were collected before and after sanitizing with Germi-X spray as shown in Fig. 5a, spread on bacteriological agar plates and allowed to grow under conducive conditions (Fig. 5b, c). Subsequently, individual colony forming units (CFUs) were counted to determine the germicidal effect of Germi-X spray and depicted as connecting plots (Fig. 5d). Average number of CFUs before sanitization was 86 [± 11.23 standard error of mean (SEM)] whereas, the count was reduced to 5.08 (± 2.06 SEM) (p < 0.001) after sanitization. Thus, sanitization with Germi-X spray reduced the germ load on smartphones by ~ 17-fold, suggesting a sizeable decrease in smartphone-mediated spread of pathogens.

## Discussion

A life without smartphone is unimaginable in today’s world. Despite conscious efforts to reduce smartphone usage owing to several proven health hazards, outcomes reveal an opposite effect more than ever. Every time the smartphone is touched, it is converted into a more potent fomite. With a frequency of 58–96 times screen contact in a 16 h active period per day (8 h being an average sleep duration in humans), we are touch our smartphone screens every 10–15 min. That is quite a huge contact time for fomite-mediated transmission of infectious diseases through phones. Plus, the situation gets really alarming if the smartphones are carried to the bathroom/toilet, which, clearly is the case as a study by the TechRepublic reveals the ‘The Dirty Truth’. A shocking 88% has adopted this gross practice, out of 44% does it invariably always and a 41% admitted that when their hands are busy, they hold their phones in mouth! Still worser, 89% carry such contaminated phones to the kitchen without sanitization (Mendoza 2019). Flushing propels germs up to 10 inches above the toilet seat (Best et al. 2012). A study from the University of Arizona showed that a median of 17,032 bacterial 16S rRNA gene copies were detected per phone from high-schoolers, which is 10 times that found on an average toilet seat (Koljalg et al. 2017).

Therefore, a more pragmatic solution is to instill a frequent smartphone-sanitizing-societal behavior. An eco-friendly sanitizer to keep our smartphones clean without burning a hole in the pocket is the key component of this solution. As it goes without much ado, herbal-based preparations are usually non-toxic, non-polluting, energy-saving with a very big ‘Go-Green’ tag that effectually runs on the premise of environment-friendliness. The germicidal herbal spray, mentioned in this study, is a humble attempt to add ‘Green’ in surface sanitization in the worldwide ‘Go-Green’ efforts. The current study was therefore designed to evaluate the disinfecting efficiency of the herbal surface sanitizer, Germi-X spray on smartphones. Additionally, its antimicrobial property was also verified microbiologically using different bacterial pathogens. In these critical times of persistent COVID-19 pandemic, wherein, contagion spreads through surface contacts, besides, aerosol, this study is a timely endeavor to offer an alternative option for managing fomite-mediated spread of infections.

The experimental evidences in favor of the germicidal efficacy of Germi-X spray against surface pathogens, like, *S. epidermidis*, *S. aureus*, *P. fluorescens* and *P. aeruginosa* are compelling. In fact, we observed that under microbiological set-up, Germi-X spray was more (6–67%) more effective in inhibiting the growth of these pathogens. Disc diffusion assay revealed that Germi-X spray was retained on the filter discs for longer duration due to which the zones of inhibition could form in case of all these pathogens. Such zone inhibition was not observed in case of standard despite the fact that it was an alcohol-based solution. In order for the standard to be effective as a germicidal, a certain minimum duration of its retention on the filter discs was required, but was not what actually happened. Being alcohol-based, the standard evaporated instantaneously from the filter disc, thus, exhibiting no inhibitory effect on the underlying bacterial lawn. It is worth speculating that if an absorbing surface, like, filter disc could not retain a standard alcohol-based surface sanitizer, how, effectively, inanimate non-absorbent surfaces would reserve. Thus, entirely alcohol-based sanitizers may be suitable for flash decontamination, suitable for surfaces with rapidly rising germ loads, such as, the ones in healthcare set-ups. For more domestic fomites, a sanitizer with prolonged bioavailability will be more effective. Smartphones, topping the domestic fomite list for germ loads, were chosen for evaluating the germicidal efficacy of Germi-X spray. *Aloe vera* being one of its constituting components, Germi-X spray had longer surface retention, with plausible implications of improved and protracted sanitization. Besides, the extracts of *A. indica* and *O. sanctum*, the two medicinal plants well known for their purifying effects on the environment account for the observed anti-microbial effect and concomitant sanitizing efficiency of Germi-X spray. Some reports show that ethanolic extracts of *A. indica* and *O. sanctum* are more effective as antibacterial agents (Rajasekaran et al. 2008). However, aqueous extracts of *A. indica* and *O. sanctum* were included in the Germi-X spray based on our previous observation that these extracts offered excellent antibacterial effects (Balkrishna et al. 2020). Sanitization of smartphones with ultraviolet C (UVC) rays is yet another method of surface decontamination that has recently gained popularity. However, spots unreachable to UVC rays remain contaminated thus, failing to make this process full-proof. Therefore, UVC sanitization is recommended as an additional step of disinfecting smartphones. With surface retention of Germi-X spray, frequency of UV sanitization can be reduced. The required frequency of sanitization of smartphones can widely vary depending on the personal hygiene regime of individuals. Regardless, with the COVID-19 pandemic around, frequent sanitization is recommended. With a frequency of touching the smartphone every 10–15 min, sanitization at least twice a day is recommendable. Due to better bioavailability, with Germi-X spray this sanitization frequency might be reduced thereby proving to light on the pocket as well.

The current study evaluated the germicidal efficacy of the herbal-based Germi-X spray using micro-broth dilution and disc diffusion assays and was found to be significantly more potent than the control sanitizer spray. In disc diffusion assay, while, the control sanitizer did not show any zone of inhibition, Germi-X spray formed distinctly clear bacteria-free zones around the disc soaked in sanitizer. This suggested longer surface retention, improved bioavailability and consequential more pronounced germicidal activity of Germi-X spray. It also demonstrated efficient disinfection of hands and mobile phones implicating preventive potentials against fomite-mediated transmission of germs. This study is a first of its kind in exploring the surface sanitization potential of an herbal spray. In a nutshell, the current study has provided compelling evidence to prove the disinfecting potential of an herbal-based surface sanitizer on smartphones, and thereby, exhibited its novelty in identifying the herbal way to manage fomite-based infection spread.

## Data Availability

All data generated during this study is included in the manuscript.

## Abbreviations

BAW: Bacterial load after wash/sanitization
BBW: Bacterial load before wash/sanitization
CFU: Colony forming units
COVID-19: Coronavirus disease 2019
HPIV: Human parainfluenza viruses
MERS-CoV: Middle east respiratory syndrome
MHA: Muller Hilton Agar
MTCC: Microbial Type Culture Collection
NCMR: National Centre for Microbial resource
SEM: Standard error of mean
TSA: Tryptic Soya Agar
UVC: Ultraviolet C
